# Safety analysis of STA-MCA bypass combined with EDAS in the treatment of patients with moyamoya disease

**DOI:** 10.1097/MD.0000000000041769

**Published:** 2025-03-21

**Authors:** Chao Zhu, Yunhong Wang, Junnan Li

**Affiliations:** a Neurosurgery Department, Pu’er People’s Hospital, Pu’er, Yunnan, China.

**Keywords:** cerebral hemodynamics, EDAS, moyamoya disease, neurological function, superficial temporal artery to middle cerebral artery (STA-MCA) bypass

## Abstract

This study evaluates the clinical efficacy and safety of superficial temporal artery to middle cerebral artery (STA-MCA) bypass combined with encephalo-duro-arterio-synangiosis (EDAS) in the treatment of moyamoya disease. A total of 80 patients with moyamoya disease who were treated at our institution between January 2022 and January 2024 were enrolled and randomly assigned to 2 groups: a control group (n = 40) and an observation group (n = 40). The control group underwent EDAS, while the observation group received STA-MCA bypass in addition to EDAS. Surgical success rates, cerebral blood flow (CBF) efficacy markers, neurological function scores, and surgical safety were comprehensively assessed in both groups. The surgical success rate in the observation group was 95.00%, significantly higher than 75.00% in the control group (*P* < .05). Three days postoperatively, the observation group exhibited significantly higher CBF and cerebral blood volume compared to the control group, with reduced time to peak and mean transit time (*P* < .05). One month after surgery, the observation group had significantly lower modified Rankin Scale and National Institutes of Health Stroke Scale scores, and higher mini-mental state examination scores compared to the control group (*P* < .05). The incidence of perioperative complications was 15.00% in the observation group and 17.50% in the control group, with no significant difference between the groups (*P* > .05). STA-MCA bypass combined with EDAS significantly improves surgical success rates, cerebral hemodynamic parameters, and neurological function outcomes in patients with moyamoya disease without increasing the incidence of surgical complications, indicating favorable safety.

## 1. Introduction

Moyamoya disease (MMD) is a rare and progressive cerebrovascular disorder, with a disease course that usually progresses over several months to years, although the speed of progression and clinical manifestations may vary between individuals. The disease is characterized by stenosis or occlusion of the distal internal carotid arteries and their main branches, including the middle cerebral artery (MCA) and anterior cerebral artery, leading to the formation of an abnormal vascular network at the base of the brain. These vascular changes appear as a “puff of smoke” on angiography, giving the disease its name “Moyamoya.” Although the exact cause of the disease is not fully understood, studies suggest that genetic factors (particularly in pediatric patients), environmental factors, and immune responses may play a role in the disease’s development.^[1,2]^

The clinical manifestations of moyamoya disease are diverse, with common symptoms including transient ischemic attacks, stroke, intracranial hemorrhage, seizures, and cognitive dysfunction. These symptoms not only severely affect the patient’s health but also significantly lower quality of life and may even be life-threatening. Therefore, early diagnosis and timely treatment are crucial for improving patient prognosis.^[3,4]^ The disease is most commonly seen in children and adolescents but can also occur in adults. Due to the insidious nature of early symptoms, diagnosis is often delayed.

The pathophysiology of moyamoya disease primarily involves the narrowing and occlusion of the main arteries of the brain, leading to insufficient cerebral blood flow (CBF). In an attempt to compensate for this reduced blood flow, an abnormal network of collateral vessels forms at the base of the brain. However, these collateral vessels often cannot adequately meet the metabolic demands of the brain. As the disease progresses, these collateral vessels become increasingly fragile, increasing the risk of both ischemic and hemorrhagic events.^[5]^

Currently, surgical treatment is considered the most effective method for managing moyamoya disease, significantly improving CBF and preventing further ischemic and hemorrhagic events. The most commonly performed surgical procedures are the superficial temporal artery to middle cerebral artery bypass (STA-MCA bypass) and encephalo-duro-arterial bypass (EDAS). Both procedures aim to restore CBF by creating new vascular channels. The STA-MCA bypass establishes a bypass by surgically connecting the STS-MCA, directly improving blood supply. The EDAS procedure involves connecting the STA to the dura mater, using arterial pulsation to stimulate the growth of new blood vessels in the dura and brain tissue, gradually improving CBF. Both procedures are minimally invasive, and most patients can resume daily activities within a short period after surgery, with lower complication risks and faster recovery compared to traditional craniotomy. Although these surgical techniques have shown good clinical outcomes in most cases, individual differences among patients may lead to varying results. Therefore, these procedures should be tailored to the patient’s specific condition and disease progression.^[6,7]^

Although these surgical techniques have been proven to effectively improve CBF and reduce ischemic events, there is no unified clinical consensus on the optimal surgical method for moyamoya disease. Some studies suggest that the STA-MCA bypass provides quicker and more direct results, while EDAS may offer more gradual improvements. Additionally, the combination of STA-MCA bypass and EDAS has been explored as a potential treatment strategy, particularly in complex cases, where combined surgery may provide more comprehensive restoration of blood flow. However, the safety and efficacy of combined surgery still lack large-scale, multi-center clinical data support.^[8,9]^

The aim of this study is to evaluate the safety and efficacy of STA-MCA bypass combined with EDAS in the treatment of moyamoya disease. We will analyze the clinical outcomes of this combined surgical treatment, including postoperative complications, recovery, and long-term follow-up results, in order to provide more empirical evidence for the surgical treatment options of moyamoya disease.

## 2. Materials and methods

### 2.1. Clinical data

This retrospective study was approved by the Ethics Committee of Pu’er People’s Hospital. Cases were reviewed based on the treatment method. A total of 80 patients diagnosed with moyamoya disease, who were admitted to our hospital between January 2022 and January 2024, were divided into 2 groups: a control group and an observation group, with 40 patients in each group. According to the patients’ main performance type, 21 patients experienced transient ischemic attacks, 14 patients had ischemic strokes, 43 patients had hemorrhagic strokes, and 2 patients had other manifestations. Additionally, 47 patients had 2 different symptoms. According to Suzuki staging, there were no patients in stage 0, 16 patients in stage 2, 43 patients in stage 3, 7 patients in stage 4, 14 patients in stage 5, and no patients in stage 6 (Table 1). The study included 40 males and 40 females, aged 21 to 61 years, with a mean age of 40.91 ± 8.93 years. In the observation group, there were 18 males and 22 females, aged 21 to 50 years, with a mean age of 40.10 ± 9.28 years. The disease duration in this group ranged from 1 to 7 months, with an average of 3.89 ± 1.06 months. In the control group, there were 22 males and 18 females, aged 30 to 61 years, with a mean age of 41.58 ± 8.98 years. The disease duration in this group also ranged from 1 to 7 months, with an average of 4.38 ± 1.02 months. There were no significant differences in the baseline characteristics between the 2 groups (*P* > .05).

**Table 1:** Clinical manifestations of patients.

Factor	n
Gender	
Male	40
Female	40
Family history of moyamoya disease	0
Performance type	
Transient ischemic attack	21
Ischemic stroke	14
Hemorrhagic stroke	43
Others	2
Patients with 2 different symptoms	47
Suzuki staging	
1	0
2	16
3	43
4	7
5	14
6	0

Inclusion criteria: Patients diagnosed with moyamoya disease through computed tomography, magnetic resonance imaging, and digital subtraction angiography (DSA), meeting the standards in the “Chinese Expert Consensus on the Diagnosis and Treatment of Moyamoya Disease and Moyamoya Syndrome.^[5]^” patients aged 30 to 75 years who were newly diagnosed during their first hospitalization and underwent surgical treatment with corresponding indications. Patients who received health education on the main content of the study upon admission and voluntarily participated in the study. The study protocol was approved by the hospital’s ethics review board. The clinical presentations of the patients are shown in Table 1.

Exclusion criteria: Patients with other cranio-cerebral tissue injuries. Patients with contraindications to surgery. Patients who developed severe postoperative complications and were transferred to intensive care. Patients with incomplete general demographic data or missing data in the evaluation indicators.^[10]^

### 2.2. Treatment methods

#### 2.2.1. Control group

The control group underwent EDAS. Preoperative assessments were completed, and the patients received general intravenous anesthesia. Once anesthesia was effective, a linear incision was made along the course of the STA. The STA was carefully isolated, and the superficial fascia on both sides of the artery was incised and separated. After removing the bone flap, the dura mater was exposed and incised, allowing the STA to be placed on the surface of the arachnoid membrane. The dura mater was then folded back and positioned over the arachnoid membrane. After confirming proper placement, temporalis fascia was used to fill the defect in the dura mater. Finally, the surgical incision was sutured layer by layer to complete the procedure.^[11,12]^

#### 2.2.2. Observation group:

In addition to the treatment provided to the control group, the observation group also underwent STA-MCA bypass surgery. Preoperative assessments were completed, and patients received general intravenous anesthesia. Once anesthesia was effective, a modified pterional incision was made, extending posteriorly to encompass the main trunk of the STA. The main branch of the STA was carefully isolated from the skin flap, and the distal end of the STA was clipped using an aneurysm clip. The temporalis muscle was fully mobilized, with special attention given to avoid damage to the deep temporal artery network. A fronto-temporal bone flap was created around the pterional point, ensuring identification of the middle meningeal artery and its branches. The dura mater was then incised and excised according to the size and shape of the exposed temporalis muscle. The arachnoid membrane was carefully incised, and the isolated STA was anastomosed to the cortical branch of the MCA.

The anastomosis site was checked for hemostasis. After confirming there was no bleeding, the base of the temporalis muscle and the edges of the dura mater were sutured. The temporalis muscle was repositioned over the arachnoid membrane and sutured to the dura mater edges. The temporalis muscle fascia and dura mater were securely sutured together. The bone flap was repositioned over the temporalis muscle, the skull was fixed, and the skin was sutured layer by layer to complete the procedure.^[6,13]^

### 2.3. Observation indicators

#### 2.3.1. Surgical success rate:

The evaluation criteria for surgical outcomes were based on the “Chinese Expert Consensus on the Treatment of Moyamoya Disease.”^[5]^ The criteria were defined as follows:

Excellent: Restoration of normal CBF, with a reduction in MRS and NIHSS scores by more than 80%.

Good: Significant improvement in hemodynamic indicators, with a reduction in MRS and NIHSS scores by 30% to 80%.

Poor: No improvement in hemodynamic indicators, with a reduction in MRS and NIHSS scores by <30%.

The surgical success rate is calculated as (excellent + good)/ total number of cases × 100%.

#### 2.3.2. Cerebral hemodynamics:

Cerebral hemodynamic indicators, including CBF, cerebral blood volume (CBV), time to peak (TTP), and mean transit time (MTT), were measured 1 day before surgery and 3 days post-surgery using a DCU7 full digital color Doppler ultrasound diagnostic system (Xuzhou Kaixin Medical Equipment Company, Xuzhou, Jiangsu, China). Patients were positioned supine on the examination table, and the ultrasound probe was placed at the temporal window. Multiple cross-sectional views were acquired to generate 2-dimensional M-mode color Doppler images of the brain, and the relevant Doppler data were recorded.

#### 2.3.3. Neurological function scores:

Neurological function was assessed using the modified Rankin Scale (MRS), the National Institutes of Health Stroke Scale (NIHSS), and the Mini-Mental State Examination (MMSE) both before surgery and 1 month post-surgery. MRS scores range from 0 to 5, with lower scores indicating better neurological function. NIHSS scores range from 0 to 42, with lower scores indicating fewer neurological deficits. MMSE scores range from 0 to 30, with higher scores indicating better cognitive function.

#### 2.3.4. Surgical safety:

Perioperative complications, including subdural hematoma, temporalis muscle swelling, bridging artery occlusion, and hyperperfusion syndrome, were recorded and compared between the 2 groups to assess surgical safety.

### 2.4. Statistical methods

The collected data were analyzed using SPSS statistical software, version 26.0. Continuous data are expressed as mean ± standard deviation ($\bar{x}\pm s$) and were analyzed using the *t*-test. Categorical data are presented as n (%) and were analyzed using the Chi-square test (χ^2^ test). The significance level was set at α = 0.05, and differences were considered statistically significant at *P* < .05.

## 3. Results

### 3.1. Surgical success rate

The surgical success rate in the observation group was 95.00%, significantly higher than the 75.00% in the control group (χ^2^ = 4.738, *P* = .035), as shown in Table 2.

**Table 2:** Comparison of excellent rates between 2 groups of surgeries [n (%)].

Group	n	Excellent	Good	Poor	Excellent rate
Observation group	40	23 (57.50)	15 (37.50)	2 (5.00)	38 (95.00)
Control group	40	18 (45.00)	14 (35.00)	8 (2.00)	30 (75.00)
χ^2^	4.738				
*P*	.035 < .05				

### 3.2. Cerebral hemodynamic indicators

Three days post-surgery, the CBF and CBV in the observation group were significantly higher than those in the control group, while the TTP and MTT were significantly lower (*P* < .05), as shown in Table 3.

**Table 3:** Comparison of cerebral hemodynamic indicators between 2 groups (x ± s).

Group	n	CBF [mL/(min·100g)]		CBV (mL/100g)		TTP (s)		MTT (s)	
		1 d before surgery	3 d after surgery	1 d before surgery	3 d after surgery	1 d before surgery	3 d after surgery	1 d before surgery	3 d after surgery
Observation group	40	47.39 ± 5.65	62.37 ± 7.59*	3.32 ± 0.69	5.15 ± 0.92*	13.32 ± 3.97	8.49 ± 1.96*	5.38 ± 0.99	3.12 ± 0.66*
Control group	40	46.62 ± 5.75	56.92 ± 6.93*	3.41 ± 0.71	4.51 ± 0.87*	13.59 ± 4.01	10.05 ± 2.13*	5.59 ± 1.03	3.91 ± 0.80*
*t*		0.702	3.897	1.336	3.714	0.352	3.960	1.080	5.598
*P*		.484	.000	.184	.000	.726	.000	.282	.000

Compared with patients in this group before treatment.

CBF = cerebral blood flow, CBV = cerebral blood volume, MTT = mean transit time, TTP = time to peak.

* *P* < .05.

### 3.3. Cerebral angiography

In the observation group, 36 patients demonstrated significant improvement in CBF reconstruction, indicating better outcomes compared to the control group, in which 7 patients showed significant improvement. The difference in postoperative cerebral angiography scores between the 2 groups was statistically significant (*P* < .05), as shown in Table 4.

**Table 4:** Comparison of postoperative cerebral angiography effects between 2 groups.

Group	n	Significant	General	Poor	χ^2^	*P*
Observation group	40	7	20	13	11.327	<.05
Control group	40	36	4	0		

**Figure 1.**
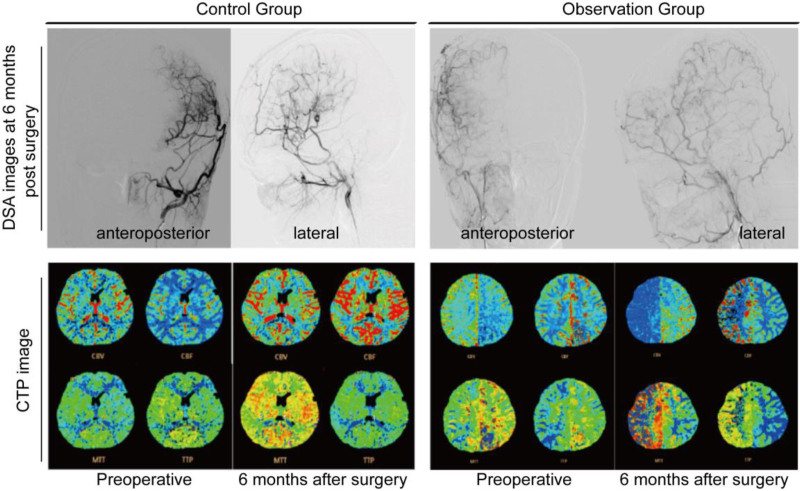
DSA before and after surgery in 2 groups of patients comparison of CTP imaging. CTP = cerebral tissue perfusion, DSA = digital subtraction angiography.

Prior to surgery, DSA revealed no compensatory blood supply from the bilateral cervical arteries to the intracranial region in either group. However, after a 6-month follow-up, significant improvement in symptoms was observed, and DSA reexamination showed good blood supply reconstruction. Comparison of pre- and postoperative cerebral tissue perfusion revealed improvements in CBF, MTT, and TTP in the bilateral fronto-temporal-vertex regions (see Fig. 1).

### 3.4. Neurological function score

One month after surgery, the MRS scores and NIHSS scores in the observation group were 0.89 ± 0.23 and 11.05 ± 2.97, respectively, both significantly lower than those in the control group, which were 1.37 ± 0.42 and 15.08 ± 3.23. The MMSE score in the observation group was 26.83 ± 3.04, significantly higher than the control group’s score of 23.75 ± 4.43 (*P* < .05), as shown in Table 5.

**Table 5:** Comparison of neurological function scores between 2 groups ($\bar{x}\pm \;s$</mathgraphic>, points).

Group	n	MRS score		NIHSS score		MMSE score	
		Preoperative	1 mo after surgery	Preoperative	1 mo after surgery	Preoperative	1 mo after surgery
Observation group	40	3.39 ± 0.87	0.89 ± 0.23*	32.41 ± 4.88	11.05 ± 2.97*	13.32 ± 3.98	26.83 ± 3.04*
Control group	40	3.50 ± 0.83	1.37 ± 0.42*	33.52 ± 5.10	15.08 ± 3.23*	19.41 ± 3.96	23.75 ± 4.43*
*t*		0.672	7.366	1.156	6.749	1.427	4.213
*P*		.503	.000	.250	.000	.157	.000

Compared with patients in this group before treatment.

MMSE = Mini-Mental State Examination, MRS = modified Rankin Scale, NIHSS = National Institutes of Health Stroke Scale.

* *P* < .05.

### 3.5. Incidence of surgical complications

The incidence of perioperative complications in the observation group and control group was 22.50% and 30.00%, respectively. There was no statistically significant difference between the 2 groups (χ^2^ = 0.581, *P* = .446), as shown in Table 6.

**Table 6:** Comparison of incidence of surgical complications between 2 groups [n (%)].

Group	n	Subdural hematoma	Temporal muscle swelling	Bridge artery occlusion	Hyperperfusion syndrome	Surgical-related acute cerebral infarction	Total
Observation group	40	1 (2.50)	1 (2.50)	2 (5.00)	4 (10.00)	1 (2.50%)	9 (22.50)
Control group	40	2 (5.00)	2 (5.00)	1 (2.50)	6 (15.00)	1 (2.50%)	12 (30.00)

## 4. Discussion

According to relevant epidemiological studies, moyamoya disease has a high incidence in clinical practice, affecting patients across various age groups. The exact etiology remains unclear; however, existing research suggests that genetic factors, intracranial vascular malformations, and arterial intimal hyperplasia are significant risk factors for the disease. As a result of intracranial arterial stenosis or occlusion, cerebral ischemia and inadequate blood supply occur, leading to symptoms such as limb weakness, speech disorders, dizziness, headaches, and impaired consciousness, with severe cases potentially progressing to shock and coma.^[14–17]^ Currently, there are no effective pharmacological treatments for moyamoya disease, and surgical vascular reconstruction is considered the most effective therapeutic option. STA-MCA bypass and EDAS are commonly used surgical techniques for treating the disease. Identifying the approach that yields better outcomes and greater safety remains a key focus of ongoing research in the surgical treatment of moyamoya disease.^[4,18]^

In this study, the observation group demonstrated a significantly higher surgical success rate, reflecting the favorable effects of combining STA-MCA bypass with EDAS. The STA-MCA bypass combines the benefits of both direct and indirect bypass techniques, effectively enhancing local CBF in a relatively short period. This approach maximizes the use of the blood supply from the carotid artery, thereby improving cerebral perfusion and establishing collateral circulation.^[19–21]^ On the other hand, EDAS involves placing vascularized muscle tissue on the surface of the brain cortex, stimulating the cortical tissue and promoting the growth of new blood vessels in the intracranial region, thereby aiding in the establishment of collateral circulation.^[22]^ Thus, the combined application of STA-MCA bypass and EDAS leverages the strengths of both techniques, achieving a synergistic effect that significantly improves the surgical success rate in patients with moyamoya disease.

In the comparison of cerebral hemodynamic indicators, 3 days post-surgery, the CBF and CBV in the observation group were significantly higher than those in the control group, while the TTP and MTT were significantly lower, indicating that STA-MCA bypass combined with EDAS can enhance CBF in patients. This combined surgical approach not only improves cerebral perfusion early on but also provides long-term vascular reconstruction through the indirect effect of temporalis muscle pasting, effectively restoring normal cerebral blood supply in a short period.^[23,24]^

In the comparison of postoperative neurological function scores, 1 month after surgery, the MRS and NIHSS scores in the observation group were lower than those in the control group, while the MMSE score was higher, indicating that STA-MCA bypass combined with EDAS can improve neurological function in patients. This improvement is attributed to the restoration of normal CBF, which effectively repairs damage to neural tissues in the brain, thereby restoring normal neurological and cognitive function.

In the comparison of surgical safety, adding STA-MCA bypass in the observation group did not significantly increase the incidence of surgical complications, suggesting that this combined surgical approach is safe.

This study has certain limitations. First, as a single-center retrospective study with a small sample size and data from only 1 hospital, the results may have limited generalizability and external validity, failing to reflect differences in clinical practices across different regions or hospitals. This could lead to negative results when assessing small differences in variables such as complications. Second, the limited follow-up period restricts the comparison of long-term efficacy. Future studies could further validate the results through multi-center, large-sample prospective designs and extend the follow-up duration to evaluate the long-term stability of treatment effects and the incidence of complications. Additionally, given regional and hospital-specific differences, future research could explore the adaptability and effectiveness of treatment strategies in different clinical settings.

In summary, the treatment of moyamoya disease with STA-MCA bypass combined with EDAS demonstrates significant efficacy. This approach improves the surgical success rate and cerebral hemodynamic indicators without increasing the incidence of surgical complications, indicating its good safety profile.

## Author contributions

**Conceptualization:** Chao Zhu, Yunhong Wang, Junnan Li.

**Data curation:** Chao Zhu, Yunhong Wang, Junnan Li.

**Formal analysis:** Chao Zhu, Yunhong Wang, Junnan Li.

**Investigation:** Chao Zhu, Yunhong Wang, Junnan Li.

**Methodology:** Chao Zhu, Yunhong Wang, Junnan Li.

**Writing – original draft:** Chao Zhu, Yunhong Wang, Junnan Li.

**Writing – review & editing:** Chao Zhu, Junnan Li.
